# Investigation on Modulation-Based Straightness Measurement

**DOI:** 10.3390/s23062912

**Published:** 2023-03-07

**Authors:** Andrea Egidi, Alessandro Balsamo, Davide Corona, Marco Pisani

**Affiliations:** Istituto Nazionale di Ricerca Metrologica (INRiM), 10135 Torino, Italy

**Keywords:** straightness, retroreflectors, high-index ball lenses, backscattering, coordinate measuring machines, machine tools

## Abstract

The concept of a novel non-contacting technique for measuring straightness and its practical realization in a mechanical device are presented in this article. The device, called InPlanT, is based on the acquisition of the luminous signal retroreflected by a spherical glass target and impinged on a photodiode after mechanical modulation. The received signal is reduced to the sought straightness profile using dedicated software. The system was characterized with a high-accuracy CMM and the maximum error of indication was derived.

## 1. Introduction

Straightness is a form characteristic of a profile [1]. The profile may be extracted either from a surface by intersection with a straightness plane (nominally orthogonal to the surface) or from a path in space by projection onto a straightness plane. The former is the case of material workpieces and standards and is relevant in inspections; the latter is the case of machines (such as machine tools or CMMs, coordinate measuring machines) and contributes to their performance. Good straightness performance of machines along their linear axes is achieved by good straightness of their guides. The performance extension to any skew line within the working volume requires good coordination of the axis motions too.

The local straightness deviation, Δ*S*_l_, is the deviation of each point of the profile from a reference line. The reference line is a matter of choice and should match the intended use of the straightness characterization. Predefined reference lines are the mean minimum zone reference line (MZ) and the least-squares reference line (LS). The reference line through two predefined points such as the profile extremes is also often used in practice as it naturally matches the alignment procedure of zeroing the instrument at the stroke extremes. Straightness parameters are derived from the local straightness deviations to characterize the profile globally, such as the peak-to-valley straightness deviation (STRt) or the root-mean-square straightness deviation (STRq).

The practical methods for measuring the straightness are based on two approaches: measuring an angle or a displacement [2].

Angle measurements are mainly used to assess the straightness of a guide, a rail, or other artifacts. The most common method consists in fixing a mirror on a square which is placed in contact with the rail being measured. The tilts of the mirror are measured with a fixed remote optical system while the square scans the length in a continuous way or in discrete steps. The tilts of the surface are reconstructed from the tilts of the mirror, hence the straightness. The instruments typically used are based on angle sensors [3], angular interferometers [2,4] and autocollimators [5]. Alternatively, a level can be used for the same purpose [6]. In the case of optically machined surfaces, the same surface can serve as a reflecting mirror achieving even more accurate results, although this seldom applies to the large scale [7]. Finally, the machine vision-based methods are worth mention and are progressively improving their performances [8].

The movement of a machine in space is along a line that is not a material and the only way to check for straightness is to measure the orthogonal displacement. This can be made in two ways: by reference to a material standard (a ball bar, a plane, etc.) [9] or to a laser beam (assumed as perfectly straight). In the first case, a straightness standard—calibrated with the above-mentioned methods—is mounted in proximity of the axis under measurement and the distance between the two is measured along the path. This can be carried out with classical displacement sensors such as mechanical probes [10], optical probes, capacitive sensors [11], or interferometers. This approach suffers from the complexity of the procedure and dependence on the straightness reference and its calibration; in some cases, the straightness of the machine and of the artifact can be separated by reversal [10]. The case of the laser beam is the most practical and is the baseline for the present work. The most common implementation is based on an interferometric arrangement in which two arms of the interferometer are split into a small angle and retroreflected by a matching two-angled mirror target. A lateral displacement of the target is transduced to an unbalance of the two arms and measured by the interferometer [12]. The disadvantages of this method are the sensitivity to yaw, the complexity and cost of the interferometric set-up, and the limited distance range. Another technique is based on the measurement of the lateral displacement of the machine to the center of the laser beam: a detector such as a CMOS sensor, a quadrant photodetector, or a position sensitive detector is attached to the moving machine to yield the lateral position of a representative point of the laser beam such as the center of mass. Alternatively, the moving machine component carries a retroreflector [13] or a slit and the detector is attached to the still machine component [14]. In the most demanding applications, the non-perfect straightness of the laser beam must be considered. Air turbulences affect the stability of the beam with a random contribution which can be reduced by averaging, while the bending of the beam due to an orthogonal gradient of the air refractivity must be carefully evaluated and corrected [15]. An alternative approach worth mentioning is based on the analysis of the transverse accelerations measured when the axis is moving at constant speed [16]. The work in this paper measures the straightness of a machine path in space with respect to a laser beam by means of a sensor measuring the lateral position of the beam retroreflected by a target attached to the machine movable component.

Independently of how the data are obtained, the algorithm to derive the overall straightness value from a dataset plays a fundamental role. This is often based on least-squares or on minimum-zone algorithms [17,18], but optimization algorithms (such as the genetic algorithm [19] and particle optimization algorithm [20]) are also recently gaining popularity.

## 2. Materials and Methods

### 2.1. Background and Motivation

Large volume metrology is of key importance to many strategic industries such as automotive, aerospace, and energy and power generation. In these sectors, manufacturing, maintenance, and safety crucially depend on metrology. Innovative tools based on cheap sensors and technologies are required by the worldwide community of researchers and industrialists. A specific field of interest is that of medium–large volume machine tools: they are in charge of machining the components of large volume products and their accuracy is key.

**Figure 1:**
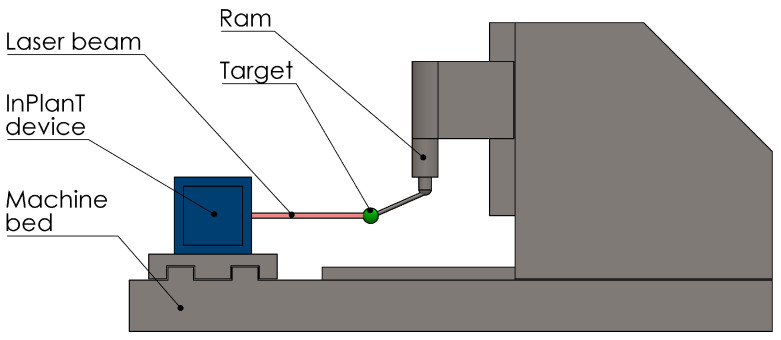
Schematic of a straightness measurement of a machine tool.

This paper describes the activity conducted at the INRIM in the European project EMPIR-17IND03 “LaVA” [21] and stems from that in the previous European project EMRP-IND53 “LUMINAR” [22]. In that project, INRIM developed a device (named “*InPlanT*”, intersecting planes technique, [23]) capable of measuring the spatial coordinates of a spherical retroreflector without contact. The motivation of the work in this paper is to adapt the InPlanT technique for measuring the geometry errors of medium/large volume machine tools (the extension to other Cartesian machines such as the CMMs is possible) and specifically its straightness errors (Figure 1). In particular, we wanted to use a retroreflecting sphere as a moving target because of its isotropy in space enabling observations from different directions. This work is an investigation of a new principle and device based on mechanical modulation. It resulted in the realization of a novel instrument capable of measuring straightness with a maximum error of ±4.8 µm over 1.2 m.

### 2.2. Concept and Theory

The pattern and phase of luminous signals retroreflected by a special target (*n* = 2 glass sphere) were studied in depth in [22,24]. Two major limitations were met:

- The distance of the target sphere from the instrument significantly affected profile and size of the retroreflected pattern;
- The accurate digital processing of the received image was time consuming and subject to ambient noise.

A new concept was introduced in this work to overcome these limitations. To speed up the measurement process and overcome the difficulties in reducing the collected image, the camera was replaced by a photodiode integrating the total intensity. To improve the resolution, a mechanical modulation was introduced by chopping the laser beam with a slit.

**Figure 2:**
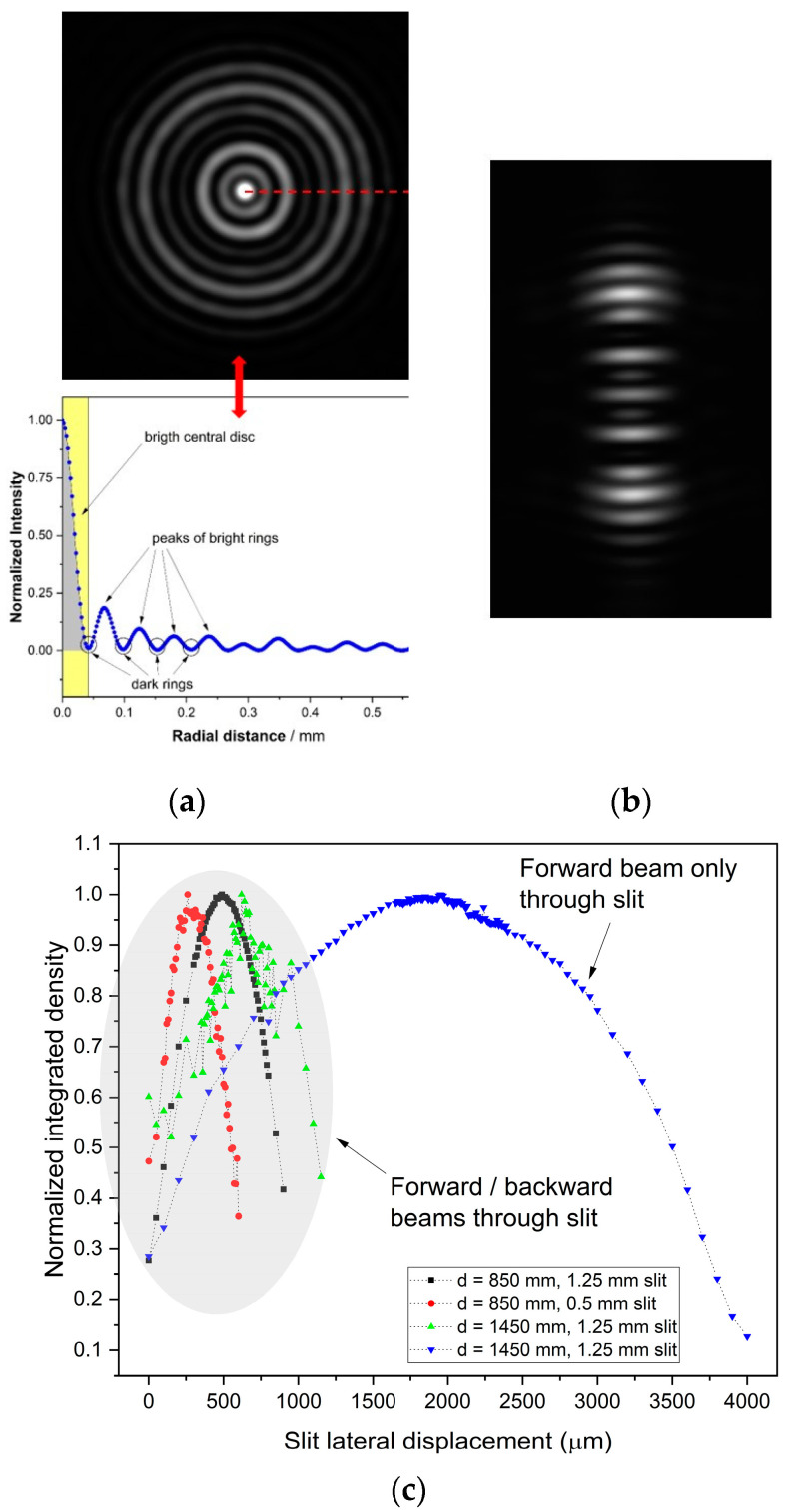
Typical patterns retroreflected from the *n* = 2 sphere (see [24] for a detailed description of the backscattering patterns from high-index glass balls) before (**a**) and after (**b**) being chopped by a rectangular 2 mm slit. (**c**) Photodiode signals with different set ups.

The target is illuminated with a collimated laser beam, which constitutes the pointing direction of the instrument and one of the directions of the straightness plane. The intensity profile of the retroreflected beam exhibits a central symmetry with a distinct peak in the center (Figure 2a). This peak propagates along the line from the target center parallel to the instrument-pointing direction and conveys the information of the target lateral position. The beam is chopped by a rectangular slit that moves along a line orthogonal to the pointing direction. The total intensity of the beam let through is integrated by a collimating lens and measured by a photodiode. When the slit position is centered on the beam peak, the photodiode signal exhibits a maximum. When the slit moves apart, the signal decreases, thus generating a signal peak corresponding to the beam peak. The position of the target sphere is then detected by the slit position at the photodiode peak. The line of movement of the slit constitutes the second direction of the straightness plane.

The backscattered image and the total power were investigated both by experiment and by simulation. The goal of the investigation was to determine the characteristic response of the photodiode to later movements of the target sphere. Figure 2 shows typical patterns of the beam before (**a**) and after (**b**) chopping by a rectangular 2 mm slit; (**c**) shows the photodiode signals with different set ups. The signal in blue is obtained when only the forward beam is chopped, and the others are obtained when both the forward and return beams are chopped. The signals in this latter set up are clearly more peaked and suitable to detect the beam center of symmetry.

**Figure 3:**
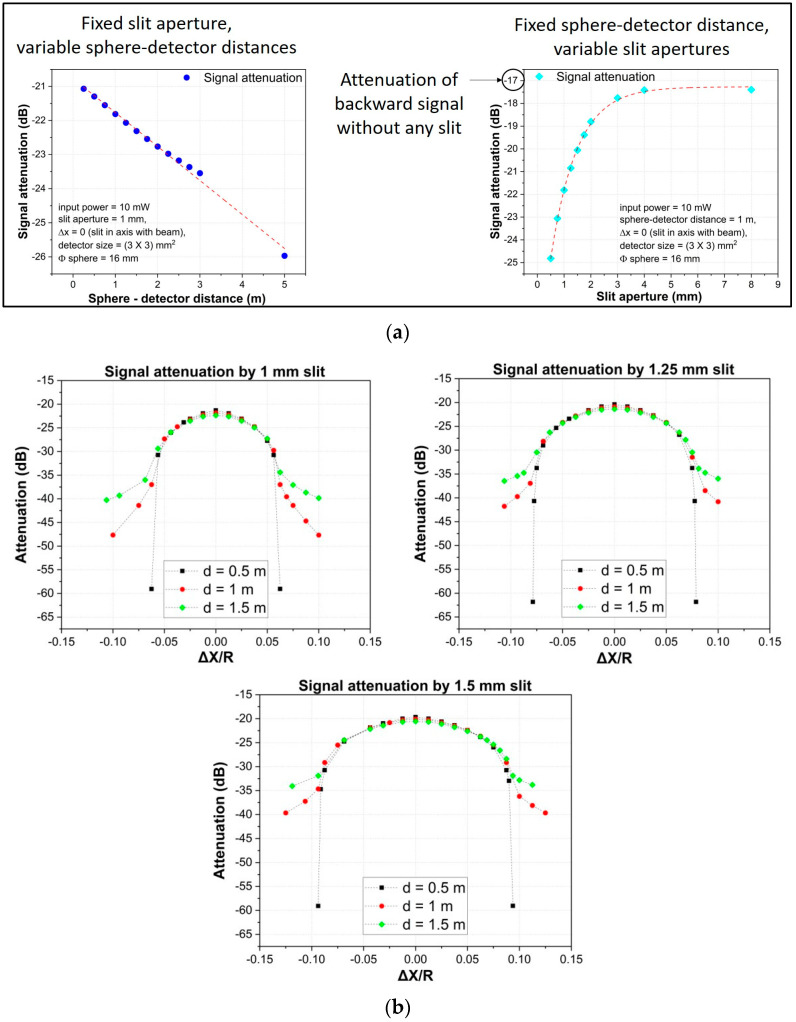
Simulated (Zemax Optic Studio software [25]) total intensity of the signal: (**a**, left) as a function of the target distance with null lateral displacement; (**a**, right) as a function of the slit width with the target at the distance of 1 m; (**b**) as a function of the sphere lateral displacement with different slit widths at three working distances of the target, 0.5 m (black), 1 m (red), and 1.5 m (green). The lateral displacement is normalized to the sphere radius *R*.

Two parameters were identified as influencing the signals: the distance to the sphere target and the slit width. To investigate them, the response was simulated in various set ups: Figure 3 shows the results. (a) Shows the total intensity received by the photodiode. On the left, with a fixed slit width at different target distances, the attenuation is linear to the distance with a rate of 1 dB/m. On the right, at a fixed distance with different slit widths, the intensity saturates to the value obtained with no slit. (b) Shows the response to the lateral movement of the sphere with different slit widths, at different distances to the sphere. With any width, the response remains the same at different distances over a wide range of lateral positions. Based on this evidence, slit widths in the range of (1–2) mm was considered suitable for this purpose.

To improve the resolution and noise rejection, a mechanical modulation was introduced. This moved from the purely static signal processing in the *space* domain to a dynamic signal processing in the *time* domain. The periodic nature of the mechanical modulation reduced the analysis to a phase measurement, with enhanced rejection of several influence factors. Furthermore, averaging over many modulation cycles was possible and easy, enabling noise compression.

**Figure 4:**
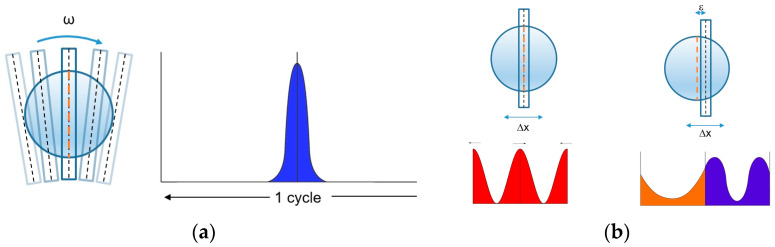
Schematic of the signals (as a function of time): rotating disc (**a**) and oscillating stage (**b**).

Two mechanisms were investigated and realized to implement the slit movement: a rotating disc and an oscillating stage. The two mechanisms were completely different in design and required very different components. More interestingly, they generated different signals: a peak per cycle with the rotating disc and two peaks per cycle with the oscillating stage (Figure 4).

In either case, the sought information conveying the target lateral position was the peak phase, within the cycle for the single peak, in reference to each other for the two peaks.

## 3. Results and Discussion

### 3.1. First Prototype (“P1”, Rotating Disc) —Description and Characterization

**Figure 5:**
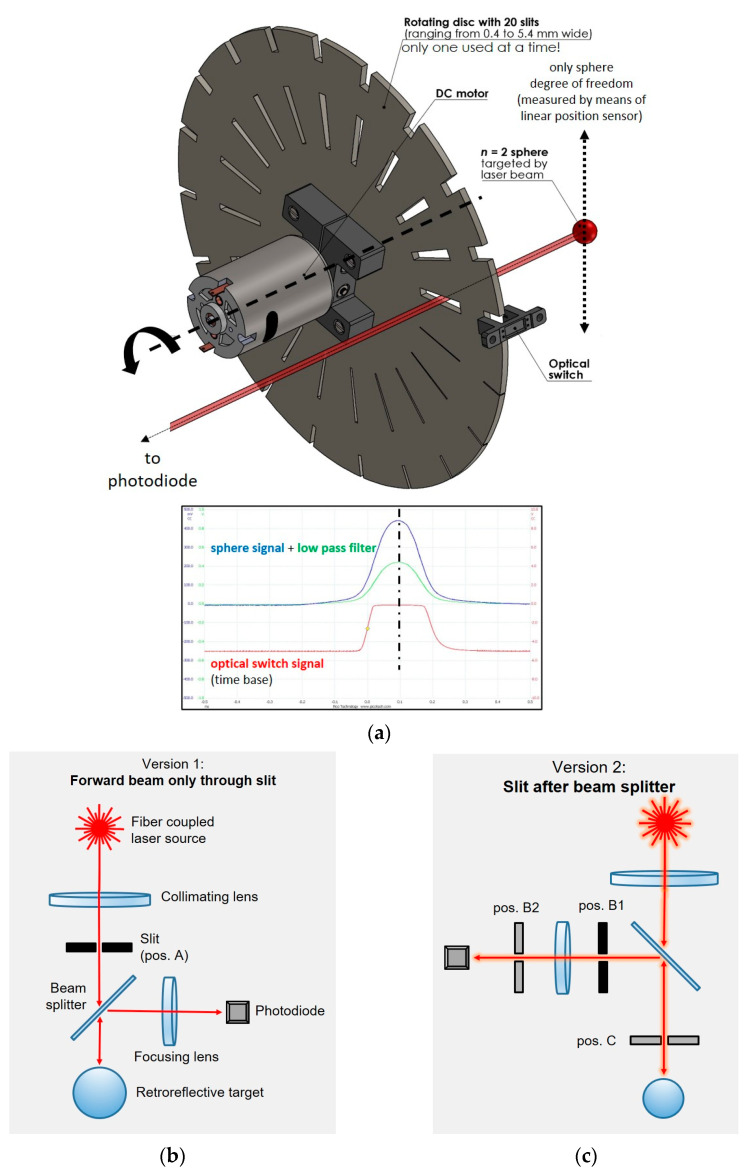
Rotating disc concept and related signals (**a**); layout A (**b**); layouts B1, B2, and C (**c**).

The first prototype was implemented based on a simple collimated laser diode as the light source, the optics to focus the retroreflected beam on the sensor (a commercial Newport photodiode), a Ø = 200 mm plastic rotating disc driven by a DC motor, and an Optek OPB815WZ optical switch at the edge of the disc generating the reference signal. The signals were acquired by a Picoscope 4424. The disc was designed with many slits of different widths to investigate their effect, but eventually one only was used (Figure 5a).

Four chopping schemes were explored, labeled A, B1, B2, and C in Figure 5b,c with reference to the position of the slit in the layout. B1 and B2 chopped the retroreflected (backward) beam only, while A and C chopped the illuminating (onward) beam as well. 

**Figure 6:**
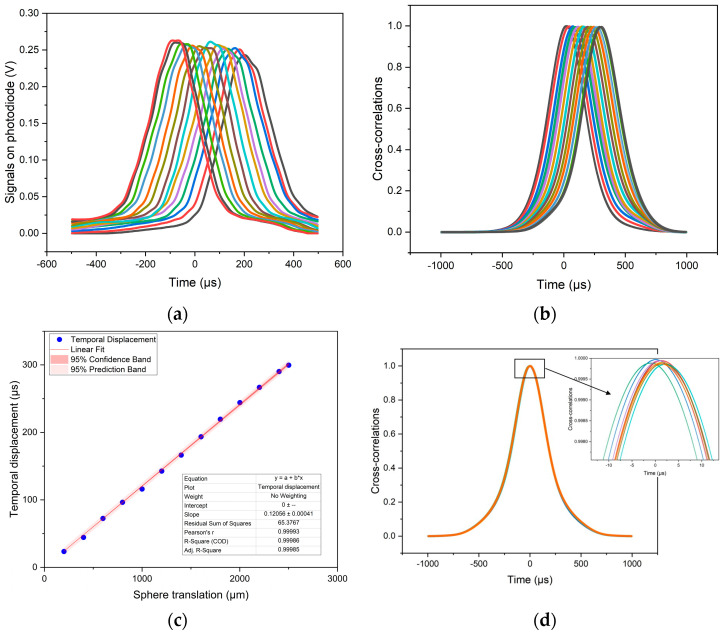
Evaluation of the resolution, rotating disc.

The rotation of the disc was detected by the optical switch independently of the target position. Its signal was taken as the time reference for the phase measurement. The photodiode detected a single peak per cycle and its phase conveyed the sought information of the target’s lateral displacement. The phase was measured by cross-correlation of the detected peak with a synthetic peak in phase with the digital signal (software developed in Python). The resolution of the system was evaluated as follows. A target was moved laterally in controlled steps of 200 µm by means of a micrometric stage and the signals were acquired (Figure 6a). Their cross-correlations with a reference signal were evaluated (Figure 6b) and the response characteristic was best fit, resulting in the slope of *k* [µs/µm] (Figure 6c). To evaluate the noise, ten signals were acquired with a still target and the standard deviation $\sigma_{t}^{\mathrm{noise}}$ [µs] of the position of their cross-correlations maxima was evaluated (Figure 6d. The close-up shows the cross-correlation curves of repeated measurements). The resolution was finally evaluated as $\sigma_{t}^{\mathrm{noise}}/k$ [µm]. Table 1 reports the results achieved with the different layouts. B1 scored the best resolution: 7.6 µm at a target distance of 145 cm.

### 3.2. Second Prototype (“P2”, Oscillating Slit)–Description and Characterization

Two limitations were met with P1: the overall size and the achievable resolution. The rotation of the slit when chopping the beam was undesired, ideally it should have been a pure translation. To minimize the rotational effect, a large disc diameter was necessary (∅ 200 mm). The instrument was blind over most of the rotation period, it detected a signal only when the slit chopped the beam, which was a tiny time fraction. This required either very fast signal detection or a low rotation speed, with an unavoidable trade-off between resolution and throughput. A new design was then introduced, whereby the modulation was realized by an oscillating—rather than rotating—slit. 

**Figure 7:**
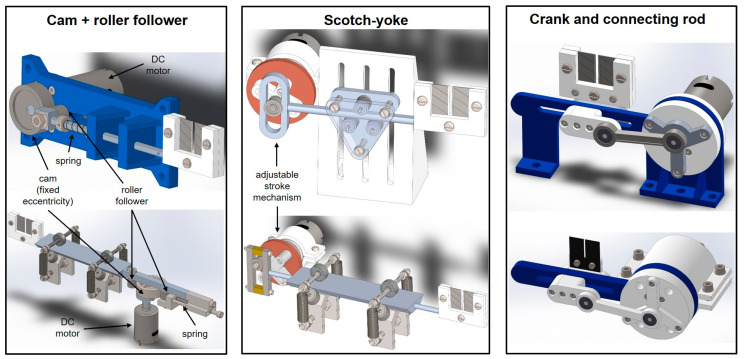
Design of reciprocating mechanisms.

Three mechanisms to realize a reciprocating motion were investigated [26]: cam and follower, scotch yoke, and the slider-crank. Tailored solutions for each one were designed (Figure 7). The crank mechanism was finally chosen as the most practical to realize.

**Figure 8:**
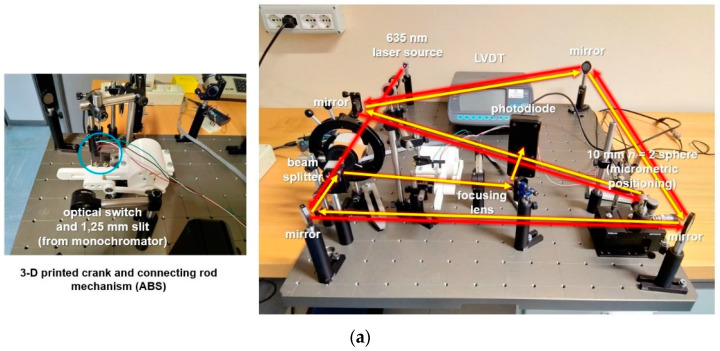

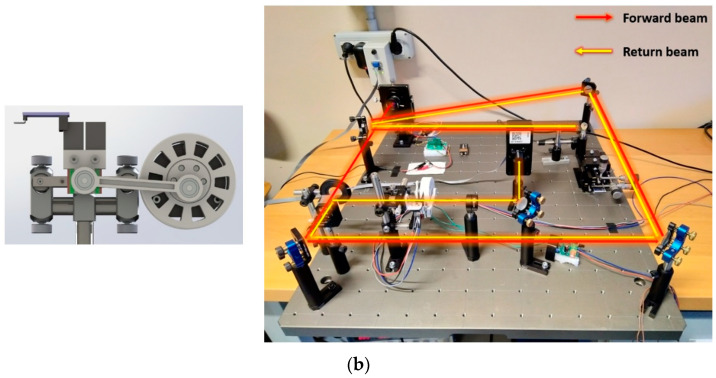
First ABS 3D-printed prototype of the slider-crank mechanisms and its test bed (**a**). Second prototype and its test bed (**b**).

The first 3D-printed ABS prototype with a DC motor, a connecting rod, two bearings, and a guide was followed by a second one with a lubricated high-quality guide and a small and accurate hard disc motor (Figure 8). They were tested on a test bed that bent the beam with repeated reflections on mirrors to achieve a 3.1 m path on a confined and portable breadboard.

While P1 yielded a peak per cycle, P2 yielded two, while the slit crossed the beam center of symmetry in either direction. The derivation of the displacement was not intuitive and required some modeling.

Let $t_{1}$ and $t_{2}$ be the time instants of the first and second peak, respectively, $t_{0}$ the initial time, T the period, and $\varphi_{1}$ and $\varphi_{2}$ the phases of the peaks. It holds:

(1) $$\varphi_{1}=2\pi \frac{t_{1}-t_{0}}{T};\;\varphi_{2}=2\pi \frac{t_{2}-t_{0}}{T},$$

(2) $$\Delta \varphi =\varphi_{2}-\varphi_{1}=2\pi \frac{t_{2}-t_{1}}{T},$$

Let y be the lateral position of the beam with respect to the central point of oscillation. The two peaks occur when the slit is at the same position, namely, that of the retroreflected beam center of symmetry. The solving equations are then:

(3) $$\left\{\begin{array}{l} y=f\left(\varphi_{1}\right)\\ y=f\left(\varphi_{2}\right)\\ \Delta \varphi =\varphi_{2}-\varphi_{1} \end{array}\right.$$

where f is the law of motion of the slit as a function of the phase. The cycle can be divided into two intervals where f is monotonic (either increasing or decreasing). $\varphi_{1}$ occurs in the first interval and $\varphi_{2}$ in the second. Let us introduce the piecewise inverse functions g and h to simultaneously solve Equation (3).

(4) $$\left\{\begin{array}{l} \varphi_{1}=g\left(y\right)\\ \varphi_{2}=h\left(y\right)\\ \Delta \varphi =\varphi_{2}-\varphi_{1} \end{array}\right.\;\;\Rightarrow \;h\left(y\right)-g\left(y\right)=\Delta \varphi$$

Equation (4) is the sought equation to derive the target position y as a function of the phase difference Δφ of the two peaks. Let us now consider the sensitivity s:

(5) $$s=\frac{\partial \Delta \varphi }{\partial y}=\frac{dh\left(y\right)}{dy}-\frac{dg\left(y\right)}{dy}=\frac{1}{\frac{df\left(\varphi_{2}\right)}{d\varphi_{2}}}-\frac{1}{\frac{df\left(\varphi_{1}\right)}{d\varphi_{1}}}$$

By introducing the slit speeds $v_{1}$ and $v_{2}$ at the peaks and considering the definition of phase, we get:

(6) $$s=\frac{1}{v_{2}\frac{dt_{2}}{d\varphi_{2}}}-\frac{1}{v_{1}\frac{dt_{1}}{d\varphi_{1}}}=\frac{1}{v_{2}\frac{T}{2\pi }}-\frac{1}{v_{1}\frac{T}{2\pi }}=\frac{2\pi }{T}\left(\frac{1}{v_{2}}-\frac{1}{v_{1}}\right)=-\frac{4\pi }{v_{H}T}\;\frac{1}{v_{H}}=\frac{1}{2}\left(\frac{1}{v_{1}}-\frac{1}{v_{2}}\right)$$

where $v_{H}$ is the harmonic mean of the speeds at the peaks ($v_{1}$ and $v_{2}$ have opposite signs). The sensitivity is inversely proportional to the slit speed when crossing the beam. In a pure harmonic (sinusoidal) motion and with a centered target, $v_{1}=-v_{2}=A\omega$, A is the oscillation amplitude and ω is the angular speed. This shows a necessary trade-off between sensitivity and measuring interval: given an oscillation frequency, the higher the amplitude, the larger the measuring interval but the smaller the sensitivity. The actual motion is not purely harmonic: it is not harmonic *in theory* in a crank and connecting rod device and *in practice* due to mechanical imperfections. As the exact equation of motion is unknown, the device response cannot be derived from Equation (4), rather it remains to be calibrated.

**Figure 9:**
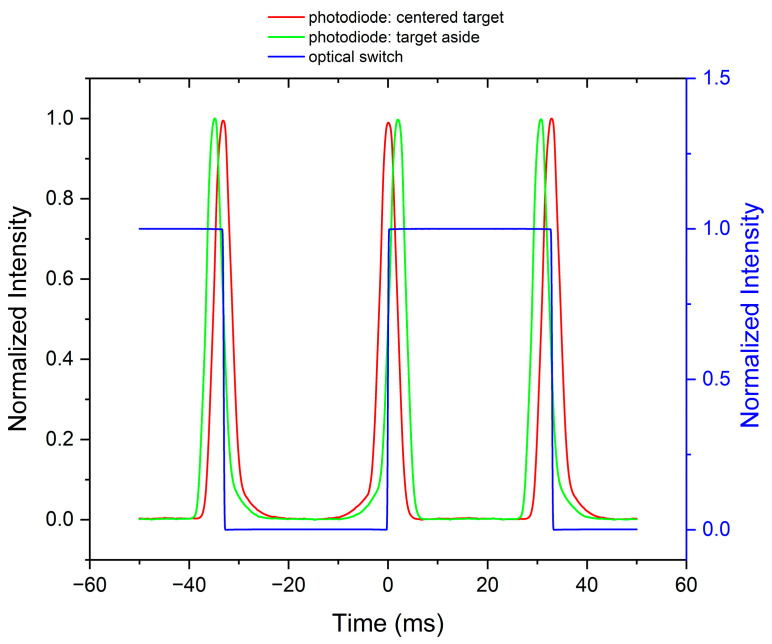
Normalized signals observed with P2 (Picoscope 4424, mean of 100 samples @ 1 MHz). Photodiode signals: target at central position (red) or 1 mm aside (green); digital switch (blue).

Figure 9 shows the normalized signals observed with the P2 device. The time base was given by a digital switch setting the phase scale (blue trace). The photodiode signal was recorded in two positions of the target: at central position (red trace) and aside (green trace). Let us call the peaks on the descending fronts of the digital signal “odd” (slit moving left) and those on the raising fronts “even” (slit moving right). When the target moved aside, the odd and even peaks shifted in opposite directions: the sequence of peaks carried phase information. This phase was derived by mutual cross-correlation. The identification of odd and even peaks—which sets the sign of the instrument indication—was made on the observation of the digital signal. Some caution was necessary when the target position was far off its central position, i.e., at the extremes of the measuring interval. The peaks tended to lose separation and to touch each other at their tails, particularly at large distances. Thresholding and filtering were applied as a countermeasure.

**Figure 10:**
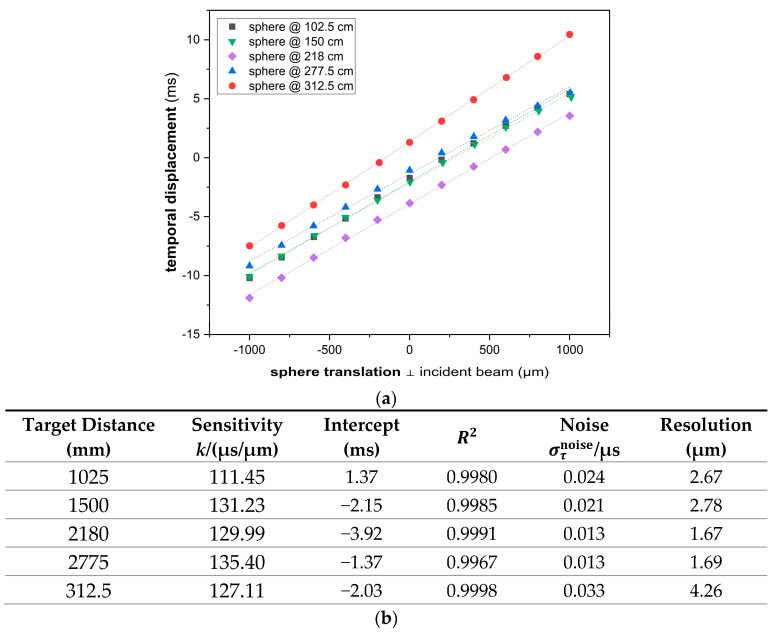
(**a**) Calibration curves at different distances to the target. (**b**) Calibration results. Lateral position of the target measured by a TESA TESATRONIC TT60 LVDT.

The resolution was investigated similarly to P1. The best achieved was 1.67 µm at a target distance of 218 cm (Figure 10), which was significantly smaller than P1.

### 3.3. Third Prototype (“P3”, Portable Device)–Description and Characterization

**Figure 11:**
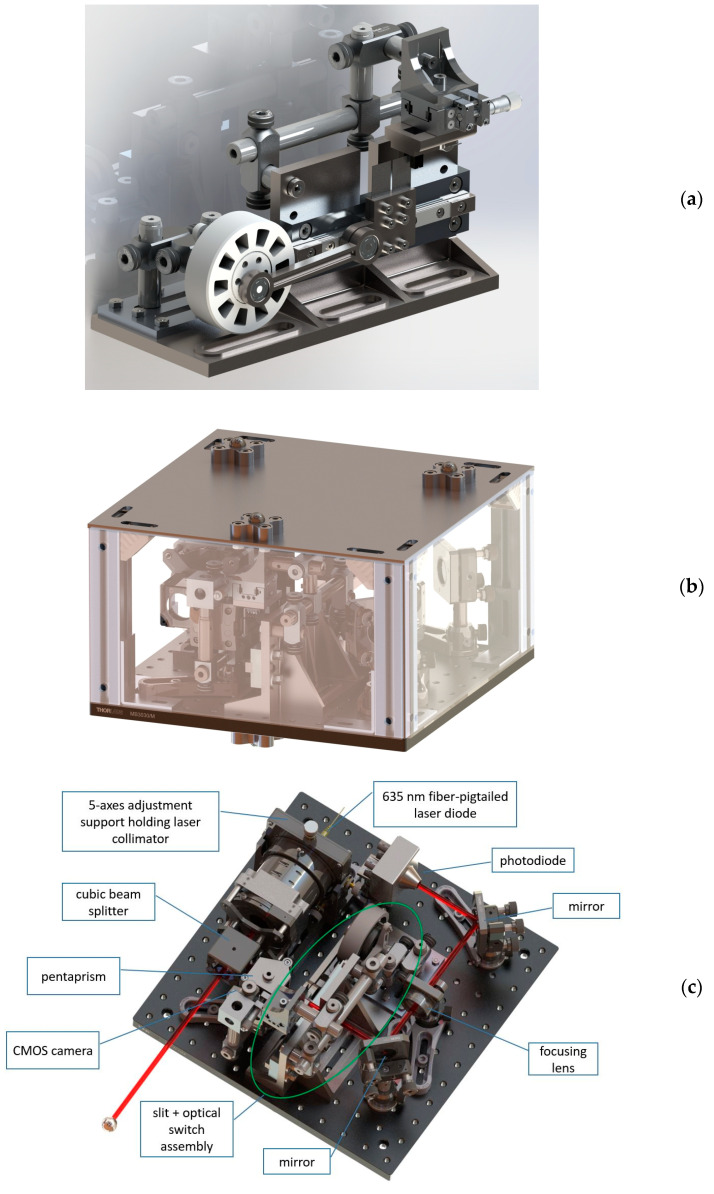
Details of the P3 design. The slider holds the oscillating slit and the digital optical switch (**a**). The overall device is enclosed with acrylic glass panels. Kinematic mounts are available on the top and bottom surfaces to enable reversal if required (**b**). Interior mounted on a breadboard (**c**).

The third prototype was developed to upgrade P2 to a compact and portable design suitable for application on machine tools. The components were scaled down and assembled as compactly as possible with care of keeping the overall design sturdy, but the ultimate compactness was not possible as P3 was still a prototype for investigation. P3 was made of aluminum machined parts, Thorlabs steel stands and clamps, and 3D-printed components (Figure 11). The degrees of freedom required for alignment and fine adjustment were identified and fine positioning facilities were introduced even beyond the expected needs for the sake of redundancy. A high-precision translation stage was used to achieve a nearly perfect 50% duty cycle of the clock signal.

P3 was tested more thoroughly than P1 and P2. As with any other straightness measuring instrument, P3 compared a measured profile with a reference line. The P3′s reference line was the light of the illuminating and retroreflected beams. Physics ensures that light rays are straight to a high degree of accuracy. Transversal gradients of the refractive index of air bend the light with effects on straightness proportional to the distance squared. This influence factor depends on the environment rather than on the instrument and is considered negligible in this application. The returned peaks changed their shape with the target distance and any sensitivity of the signal processing would result in a systematic error—effectively bending the P3 reference line. The straightness of a nearly-perfect straight stroke was measured and any measured straightness error was attributed to a systematic error of the instrument reference line.

**Figure 12:**
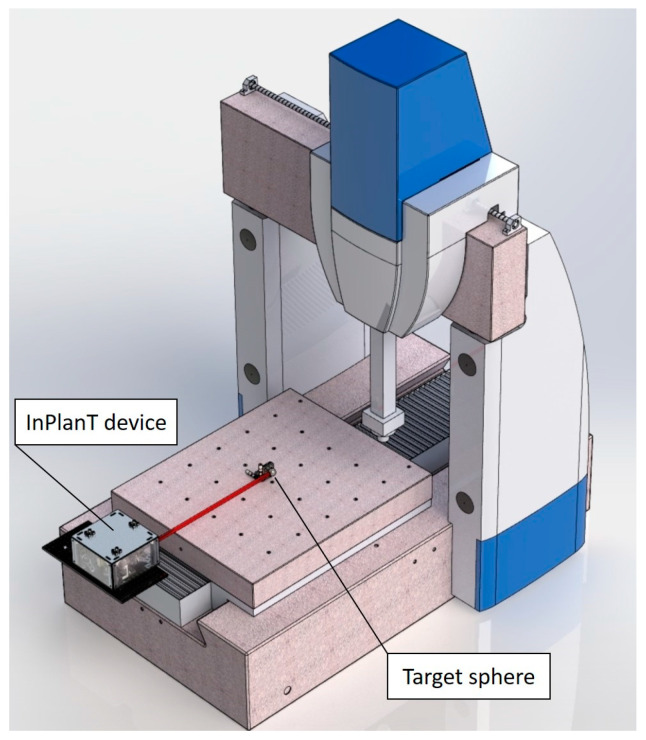
Schematic of the testing set up of the P3 on the CMM.

**Figure 13:**
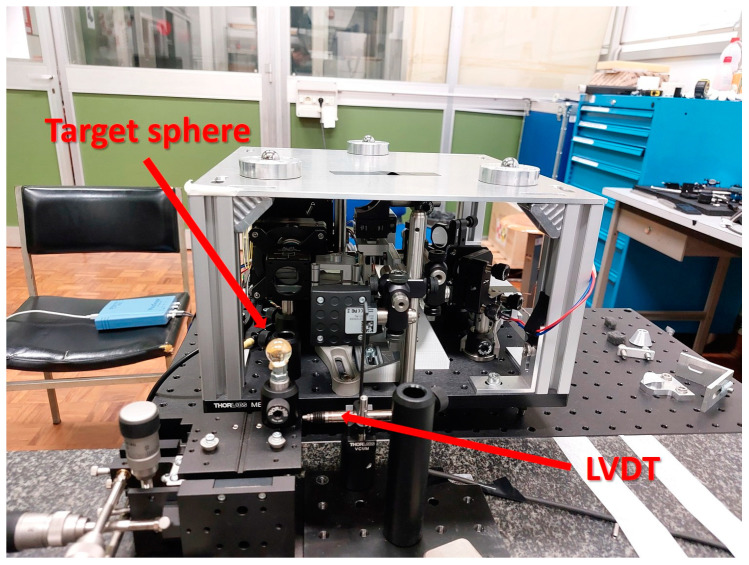
Front view of the physical implementation of P3 mounted on the CMM at INRIM.

A high-accuracy CMM available at INRIM (Leitz PMM-C 10 12107) was used for validation. Its longest stroke was 1.2 m, which imposed the maximum distance of the target. The instrument was attached to the CMM basement and the target was attached to a movable table. The target was mounted on a transversal micrometric stage whose position was measured by a precision LVDT (Figure 12 and Figure 13).

**Figure 14:**
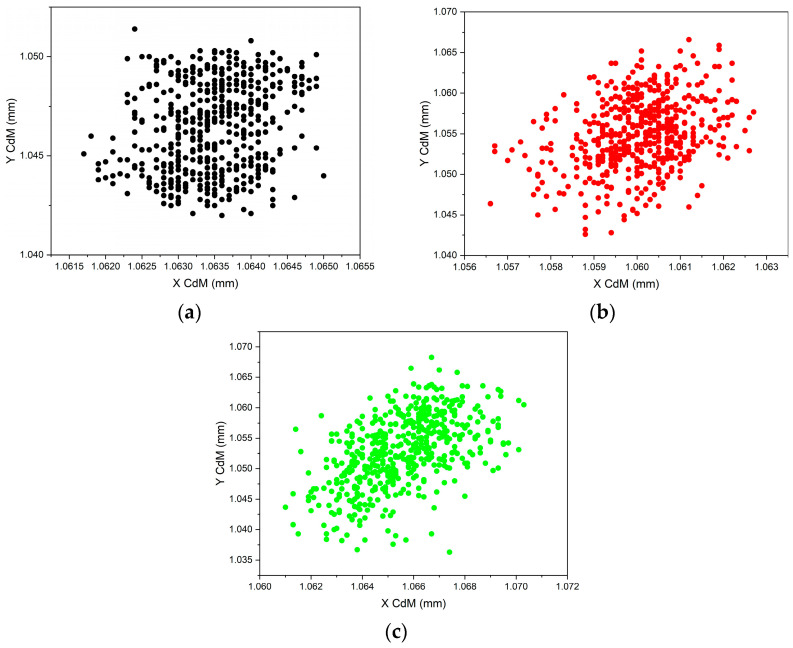
Noise and turbulence detection. Coordinates of the image center of mass (labeled “CdM”) with a still CMM at different distances *d* of the target. (**a**) *d* ≈ 0 mm (black); (**b**) *d* = 600 mm (red); and (**c**) *d* = 1200 mm (green).

A preliminary investigation was carried out of the combined effects of the vibrations caused by the slider-crank mechanism and of the air turbulence in the laboratory. A CMOS camera was added and integrated into the device to observe the return pattern. Fluctuations of a few micrometers were detected (Figure 14), indicating that averaging was required, at the cost of a reduction in the instrument throughput.

The alignment of the instrument to the CMM stroke was achieved by holding a fixed flat object (such as a piece of paper) beyond the target sphere and observing the halo generated by the external portion of the illuminating beam. When no relative movement of the halo was observed over the whole stroke, the instrument was deemed as aligned.

**Figure 15:**
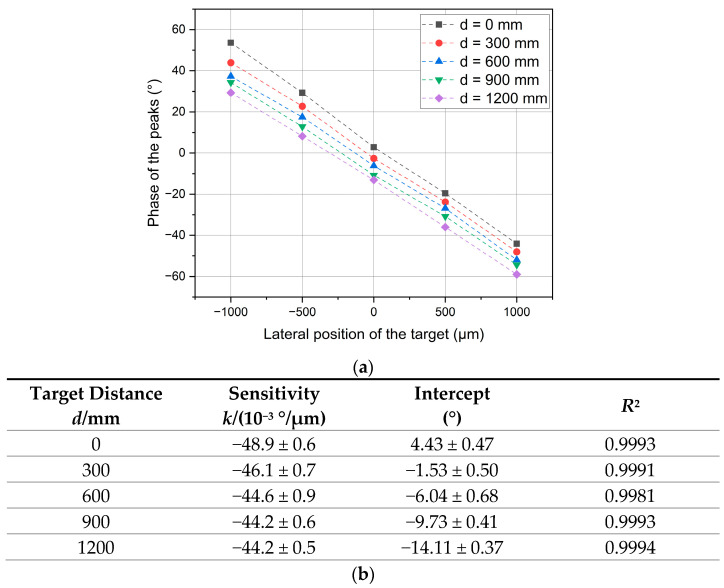
Calibration of P3: calibration curves at different distances to the target (**a**) and results of the linear best-fitting (values and standard deviations). (**b**) The time separation of the peaks is expressed as a phase (unlike in Figure 10a (P2), which is expressed as a time interval).

To transform the phase measurement to a lateral displacement measurement, a calibration was carried out. The indication of the instrument was recorded at 5 lateral positions of the target 0.5 mm apart from each other to cover a range of ±1 mm and a linear response was derived by the least-squares best-fit method. This was repeated at 5 distances to the target in steps of 300 mm to cover the available range (0–1.2) m, resulting overall in a (2 × 1200) mm^2^ grid of points. Figure 15 shows that the sensitivity was stable over the full range of distances to the target, with the exception of *d* = 0, which is slightly higher. Even if essentially parallel to each other, the calibration lines were not coincident, rather they were spread vertically. A possible explanation is a slight misalignment of the onward beam to the CMM stroke. In addition, the return beam retroreflected by the target sphere is known to be slightly divergent [24] even with a perfectly collimated forward beam: there is no single direction in the return beam.

These calibration values were then used in actual measurement. Given a distance to the target, the curves for the immediately longer and shorter distances in the calibration table were used to derive two lateral displacements, and the actual instrument indication was taken as an interpolation between them.

**Figure 16:**
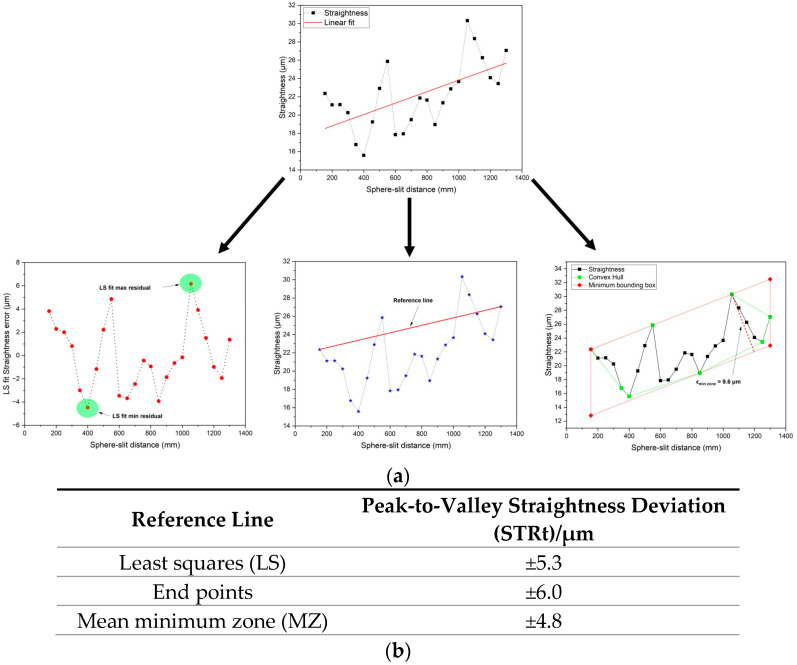
Characterization of the instrument internal reference line. Plots of the straightness vs. the distance between target and slit (**a**) and derived values of the peak-to-valley straightness deviation (STRt) (**b**).

After calibration, an independent scan of the whole stroke was carried out in 24 points in steps of 50 mm (some were 45 mm only). For each, the instrument indication was taken by interpolation as described above. Three different reference lines were considered and the consequent peak-to-valley straightness deviations (STRt) evaluated: the least-squares reference line (LS), the reference line through the extreme points, and the mean minimum-zone reference line (MZ) [27], see Figure 16.

**Figure 17:**
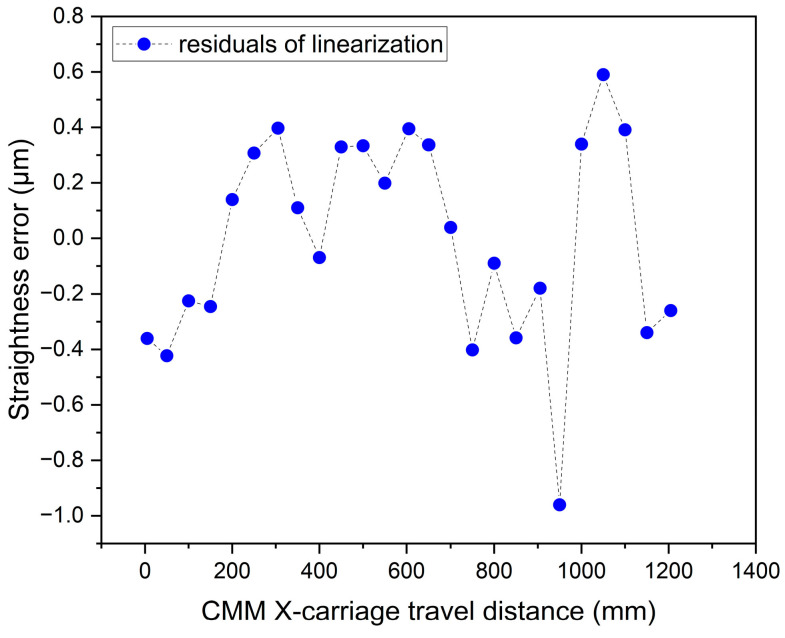
Straightness of the CMM table stroke. The least-squares reference line was subtracted and the stroke is represented as the horizontal axis.

The movement of the table from a high-accuracy CMM was taken as a reference and assumed with negligible straightness error. This assumption was based on previous long running experience in the use of the CMM. To demonstrate this, a direct measurement of the stroke straightness was performed. This mimicked the previous experiment with a laser head replacing the InPlanT device and a 5 MPix CMOS camera with 2.2 µm pixel size replacing the sphere. The straightness was measured as the center of mass of the laser spot detected by the camera. To mitigate the effect of the air turbulence, the camera filmed the laser spot at ~6 Hz for a time period of 100 s and the centers of mass of the individual stills were averaged. The position coincidence of the camera in this experiment and of the sphere in the previous one takes account of all geometry errors of the CMM combined over the path line. Figure 17 shows the result. While the first portion of the stroke shows stable and realistic results, the last portion farthest to the laser source exhibits oscillations due to the residual effects of the air turbulence. Repetitions showed that this last portion is not stable. Taking that into consideration, the straightness was assessed within ±0.5 µm, which supports the assumption of a stroke nearly perfect for the purpose. No corrections were introduced to the results in Figure 16 in view of the turbulence effects.

## 4. Discussion

The following improvements are foreseen in future work:

- A better realization of the crank and connecting rod mechanism. The current one was 3D printed in ABS and suffered slight backslash, which likely affects the repeatability.
- The projections along the onward beam direction of the beam expander center and of the slit central point were made to not coincide to the same accuracy as the other alignments. When the halo around the target sphere was centered, the two peak signals received from the photodiode were not equidistant. An improvement of these alignments is expected to improve the spread of the calibration lines shown in Figure 15a.
- Even if the instruments can easily measure at much longer distances to the target, the results were demonstrated up to 1.2 m, whereas the range up to (2–3) m is of interest for the targeted application to the machine tool. The device was designed and equipped with kinematic mounts on its upper and lower covers (not used so far). This enables the reversal technique to separate the errors of the instrument from that of the stroke, which are unlikely to be error free over a distance in excess of 1.5 m.
- The instrument is sensitive to a direction only (1D), e.g., in a horizontal straightness plane, whereas the straightness of a path in space is 2D in fact. This requires rotating the instrument 90° and repeating the procedure to achieve a full measurement. This limitation is due to the oscillating modulation, which is difficult to extend to 2D. Alternative designs of the mechanical modulation can be investigated to overcome this limitation.

## 5. Conclusions

An instrument for measuring the 1D straightness of a path in space was designed, manufactured, and tested. Two preliminary prototypes (P1 and P2) were realized before the current most-advanced prototype (P3). The working principle is the mechanical modulation of the return beam after retroreflection on a target sphere, which allows fine detection of the beam center and then of the target position. The instrument was characterized up to 1.2 m with a high-accuracy CMM. A maximum error of indication of ±4.8 µm and good linearity over ±1 mm measuring interval were demonstrated. The instrument is (300 × 300 × 175) mm^3^ in size, which is suitable for the sizes of the targeted machine tools, and can be further miniaturized.

## Figures and Tables

**Table 1:** Results of the resolutions for the P1 prototype.

Layout	Sensitivity *k*/(µs/µm)	Noise $\sigma_{\tau }^{noise}$/µs	Resolution (µm)	Target Distance (cm)
A	0.04	0.65	15.77	135
B1	0.12	0.92	7.60	145
B2	0.03	0.86	27.55	135
C	0.13	1.05	7.87	135
B1 bis ^1^	0.09	0.79	8.46	297

^1^ Same chopping scheme as B1, but with a higher target distance.

## Data Availability

Not applicable.
